# Characterization and Functional Analysis of *FaHsfC1b* from *Festuca arundinacea* Conferring Heat Tolerance in Arabidopsis

**DOI:** 10.3390/ijms19092702

**Published:** 2018-09-11

**Authors:** Lili Zhuang, Wei Cao, Jian Wang, Jingjin Yu, Zhimin Yang, Bingru Huang

**Affiliations:** 1 College of Agro-grassland Science, Nanjing Agricultural University, Nanjing 210095, China; zhuanglili2001@163.com (L.Z.); baihe.909@foxmail.com (W.C.); 2015220003@njau.edu.cn (J.W.); nauyjj@njau.edu.cn (J.Y.); 2 Department of Plant Biology and Pathology, Rutgers University, New Brunswick, NJ 08901, USA

**Keywords:** tall fescue, *HsfC1b*, heat stress, transgenic, gene function

## Abstract

Heat transcription factors (*Hsfs*) belong to a large gene family classified into A, B, and C groups, with classes A and B *Hsfs* being well-characterized and known for their roles in plant tolerance to abiotic stresses. The functions and roles of Class C *Hsfs* are not well-documented. The objectives of this study were to characterize a class C *Hsf* gene (*FaHsfC1b*) cloned from tall fescue (*Festuca arundinacea*), a perennial grass species, and to determine the physiological functions of *FaHsfC1b* in regulating heat tolerance by overexpressing *FaHsfC1b* in *Arabidopsis thaliana*. Full length cDNA of *FaHsfC1b* was cloned and the sequence alignment showed that it had high similarity to *OsHsfC1b* with typical DNA binding domain, hydrophobic oligomerization domain, and a nucleus localization signal. Transient expression with *FaHsfC1b*-eGFP in protoplasts of Arabidopsis leaves indicated its nucleus localization. qRT-PCR analysis showed that *FaHsfC1b* responded to heat, osmotic, salt, and cold stress in leaves and roots during 48-h treatment. Physiological analysis showed that *FaHsfC1b* overexpression enhanced plant survival rate, chlorophyll content, and photochemical efficiency, while it resulted in decreases in electrolyte leakage, H_2_O_2_ and O^2−^ content under heat stress. qRT-PCR showed that endogenous *HsfC1* was induced in transgenic plants and the expression levels of heat protection protein genes, including several *HSPs*, *AtGalSyn1*, *AtRof1*, and *AtHSA32*, as well as ABA-synthesizing gene (*NCED3*) were significantly upregulated in transgenic plants overexpressing *FaHsfC1b* under heat stress. Our results first demonstrate that *HsfC1b* plays positive roles in plant tolerance to heat stress in association with the induction and upregulation of heat-protective genes. *HsfC1b* may be used as a candidate gene for genetic modification of cool-season plant species for improving heat tolerance.

## 1. Introduction

Heat stress is one of the major environmental factors that adversely affects plant growth and development. Early perception and transduction of heat stress signal in plants are mainly mediated by heat stress transcription factors (Hsfs), Hsfs play an important role in regulating plant responses to environmental stresses [1]. There are 21 Hsfs in Arabidopsis (*Arabidopsis thaliana*), 26 in rice (*Oryza sativa*) and 30 in maize (*Zea mays*) [2,3,4]. Hsfs are classified into three classes: A, B, and C. The functions of Hsfs vary among different classes. HsfA1a is reported to be a master regulator of heat shock response in tomato (*Lycopersicon esculentum*) [5]. HsfA2 is known to positively regulate plant tolerance to salt stress or osmotic stress [6], oxidative stress [7], anoxia [8] and heat stress [9,10]. HsfA2 also formed super activator heterodimers with HsfA1 to synergistically activate target heat stress genes [11]. FaHsfA2c cloned from tall fescue (*Festuca arundinacea*) was found to confer improved heat tolerance in transgenic tall fescue [12]. FaHsfA2c was later found to act downstream of abscisic acid (ABA) signaling [13]. Contrary to HsfA, members of Class B, such as HsfB1 and HsfB2b, were shown to act as repressors of the expression of heat-inducible Hsfs but to positively regulate the acquired heat tolerance [14]. Class C Hsfs have been found in many species and HsfC2 is only found in monocotyledonous plants [15]. Transcription analysis showed that *HsfC* genes were upregulated by heat stress in various plant species, such as rice [16], wheat (*Triticum aestivum*) [15], cabbage (*Brassica rapa*) [17], ponkan (*Citrus reticulata Blanco*) [18], carrot (*Daucus carota*) [19], soybean (*Glycine max*) [9] and salix (*Salix suchowensis*) [20]. In some of these species, *HsfC* genes also responded to osmotic stress, cold stress, or ABA [21]. TaHsfC2a-B in wheat activated heat protection genes and acted through the ABA-mediated regulatory pathway to confer heat protection to developing wheat grains [21]. The function of HsfC1b was only analyzed in rice in a *hsfc1b* mutant and two artificial micro-RNA (*ami*RNA) knockdown lines. OsHsfC1b had transactivation potential that can be induced by osmotic stress and salt stress [22]. The functions of *HsfC1b* gene in plant adaptation to heat stress are not well characterized.

Heat shock proteins (HSPs) are involved in heat-induced transcriptional changes [23,24]. HSPs are highly conserved proteins that are classified as high molecular-mass proteins or small-molecular-mass proteins (sHSP) [25]. Some HSPs, such as HSP70 and HSP90 are constitutively expressed, although their expression can be upregulated by heat stress, while most sHSPs, such as HSP18 and HSP22, are heat-inducible and can be found in the cytoplasm, endoplasmic reticulum, and chloroplasts [25,26,27]. The stress-protective function attributed to HSPs is as molecular chaperones, and in the case of sHSPs, also as membrane-stabilizing factors [28]. Promoters of *Hsp18.1-CL* and *Hsp26.5-P(r)* contain cis-acting heat-shock elements (HSEs), which are able to bind to both HsfA1 and HsfA2 [29], and *Hsp18.1-Cl*, *Hsp22.0-ER*, *Hsp26.5-P(r)*, *Hsp-70-b* were related to thermostability in many *Hsf* transgenic plants [10,30,31]. Other heat protection proteins such as galactinol synthase (GalSyn), FK506-binding protein (Rof), and heat-stress-associated 32-KD protein (HSA32) are also reported to be the downstream factors of Hsfs and play roles in heat tolerance [32,33,34,35].

In response to heat stress, Hsfs bind to HSEs to regulate the expression of *HSP* genes [36]. For example, HsfA1/HsfA2 superactivator complex formation precedes the recognition of HSEs in tomato to activate *HSP* gene expression [11]. Direct interaction between ascorbate peroxidase (APX1), HSE, and HSF was detected via gel shift assays in Arabidopsis [37]. Hsp70 interacts with HSF to prevent the trimerization and binding of HSF to HSE, thereby inhibiting the transcriptional activation of *HSP* genes by their Hsfs [38]. In reverse, HSPs can also influence the transcription of *Hsfs*, and Hsp70 together with HsfA1 and HsfA2 modulate the abundance of *HsfB1* [39]. The same regulation is found between *Hsfs* and other heat protection genes. For example, *TaHsfC2a* in wheat acts as a direct regulator of *TaGalSyn*, and HSE present in the promoter of *TaGalSyn* is responsible for both heat induction and transactivation by *TaHsfC2a-B* [21]. Despite the knowledge of the interactive effects of *Hsfs* and *HSP* genes on heat responses, changes in *HSPs* and the expression of other heat protection genes due to Class C *Hsfs* related to improved heat tolerance in plants are not well documented.

Tall fescue is a perennial cool-season grass widely used as forage and turf grass [40]. Heat stress is often a major problem in cool-season grasses through the duration of summer and results in declined turf quality and forage yield [12,13,40,41]. Given the current global warming situation with potentially intensified and prolonged heat stress, studying mechanisms of heat tolerance by characterizing heat-responsive genes and the related regulatory network of genes in cool-season grasses such as tall fescue is of great importance for developing heat-tolerant germplasm.

We hypothesized that HsfC1b may play positive roles in regulating plant heat tolerance and that the positive effects of HsfC1b may be associated with the induction or upregulation of heat-protective genes. Therefore, the objectives of this study were to characterize *FaHsfC1b*, a Class C *Hsf*, cloned from tall fescue, a perennial grass species, by sequence analysis and subcellular localization, and to determine the physiological functions of *FaHsfC1b* in plant tolerance to heat stress by overexpressing it in Arabidopsis and analyzing the expression patterns of selected heat protection genes conferring heat tolerance as a result of its overexpression.

## 2. Results

### 2.1. FaHsfC1b Encoded a Class C Hsf and the Gene Product is Localized in the Nucleus

**Figure 1:**
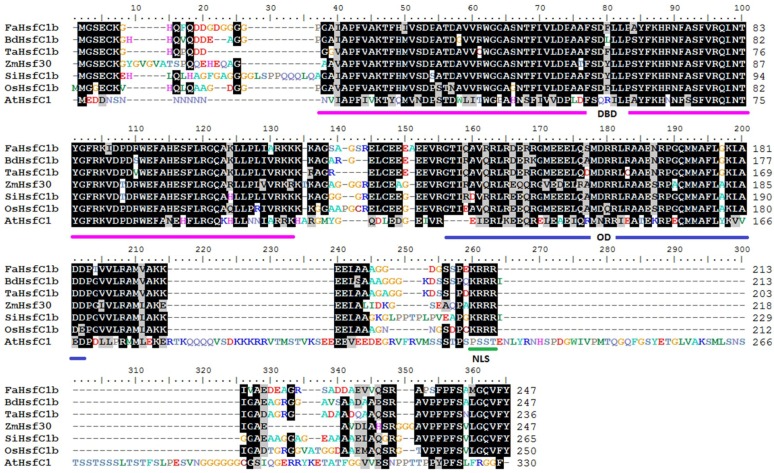
Sequence alignment of FaHsfC1b with HsfC1b from *Brachypodium distachyon*, *Triticum aestivum*, *Zea mays*, *Setaria italica*, *Oryza sativa* and HsfC1 in *Arabidopsis thaliana*. Conserved DNA binding domain (DBD), oligomerization domain (OD) and nuclear localization signal (NLS) are indicated by purplish red, dark blue and red color lines. The sequence of FaHsfC1b had been submitted to NCBI and the accession number is KY475613. Amino acid sequences of other genes were derived from phytozome (www.phytozome.net) and https://www.arabidopsis.org/index.jsp. Accession numbers of protein sequences are listed in materials and methods.

A 945-bp homology sequence was obtained by Local BLAST (‘tblastn’ program) from our transcriptome database of tall fescue using the OsHsfC1b (XP_015633152) protein sequence as the query. The blastx program showed that the protein sequence shared 73% and 75% identity with *Brachypodium distachyon* and rice, respectively. A full-length open reading frame (ORF) was predicted by the FGENESH program and a 744 bp nucleotide sequence encoding 247 amino acids was obtained. Primers were designed (Table S1) based on the predicted sequence and the fragment was amplified from the tall fescue cultivar ‘Regenerate’. The nucleotide sequence was confirmed by sequencing. Homology comparison of amino acid sequence showed that the sequence had high similarity with HsfC1b in other monocot plants but is very different from HsfC1 in Arabidopsis, especially at the C-terminal end of the protein (Figure 1).

**Figure 2:**
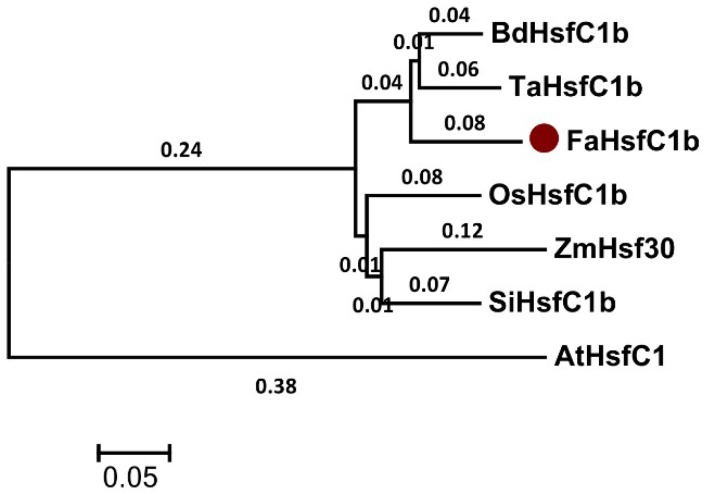
Phylogenetic tree of FaHsfC1b and other HSFC proteins. The tree was constructed by MEGA 5.1 software, based on alignment of complete protein sequences. The red dot indicates FaHsfC1b protein. Accession numbers of protein sequences are listed in materials and methods.

According to the phylogenetic tree, the sequence obtained was most closely related to the HsfC1b clade (Figure 2). Therefore, it was designated as *Festuca arundinacea HsfC1b* (*FaHsfC1b*). Characteristic domains of Class C Hsf, such as a DNA binding domain (DBD) close to the N-terminus and a short oligomerization domain (OD), also named the HR-A/B region, were found in FaHsfC1b. The nuclear localization signal (NLS) domain was located at the C-terminal of FaHsfC1b (Figure 1).

**Figure 3:**
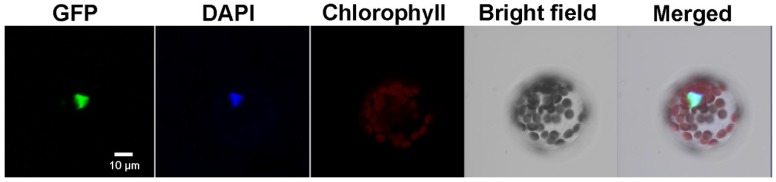
Subcellular localization of FaHsfC1b protein. FaHsfC1b-eGFP fusion protein was localized in nucleus of Arabidopsis mesophyll protoplast. Bar = 10 μm.

Transient transformation of Arabidopsis protoplasts was performed. As shown in Figure 3, the GFP signal was overlapped with the DAPI stained nucleus. This indicated that FaHsfC1b protein was localized in the nucleus.

### 2.2. Expression Pattern of FaHsfC1b Under Different Abiotic Stresses

**Figure 4:**
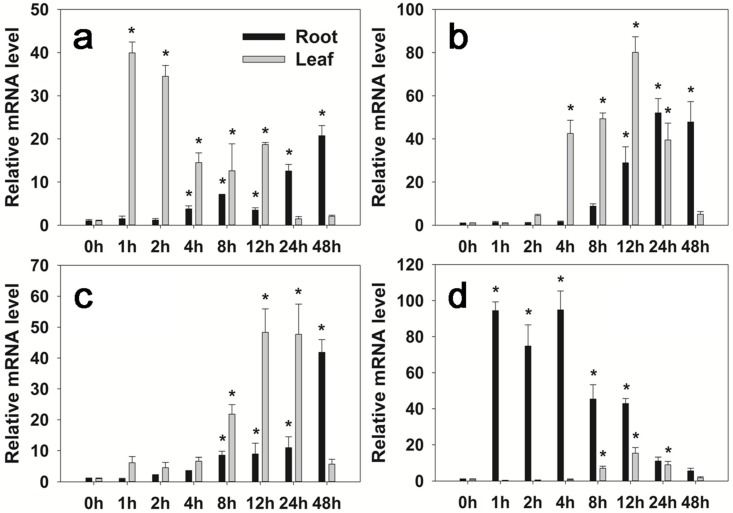
*FaHsfC1b* expression patterns in different abiotic stress conditions. Plants were treated under heat stress at 45 °C (**a**), cold stress at 4 °C (**b**), osmotic stress (20% PEG-6000), (**c**) or salt stress (150 mM NaCl) (**d**) during a 48 h period. qRT-PCR values were means ± SD of three biological repetitions (three technical repetitions for each biological repetition). Asterisks indicated significant differences of mean values at each time period of stress treatment regarding the value at 0 h (nontreated plants) for each tissue analyzed (root and leaf).

To investigate the response of *FaHsfC1b* to stresses, relative mRNA levels were analyzed by qRT-PCR. The expression levels of *FaHsfC1b* in roots and leaves increased during 48 h of exposure to heat, cold, osmotic, and salt stress (Figure 4). *FaHsfC1b* expression patterns in response to different stresses varied among different tissues. For heat treatment, the mRNA levels of *FaHsfC1b* increased in leaves and reached peak value (by 40-fold) within 1 h, while in roots *FaHsfC1b* increased in 4 h and reached the peak (by 20-fold) in 48 h (Figure 4a). Under cold stress, *FaHsfC1b* expression level increased in 4 h in leaves and in 12 h in roots, and the gene expression level in leaves reached the peak (by 80-fold) in 12 h in leaves and in 24 h in roots (by 52-fold) (Figure 4b). Under osmotic stress, *FaHsfC1b* expression level elevated in 8 h and reached the highest level at 12 h (by 48-fold) in leaves and 48 h in roots (by 41-fold) (Figure 4c). For salt stress (Figure 4d), the expression of *FaHsfC1b* in roots increased in 1 h of treatment and reached the peak (by 94-fold) in 4 h. Leaf *FaHsfC1b* expression level increased in 8 h and reached the peak (by 19-fold) in 12 h. At 48 h, the mRNA level of *FaHsfC1b* in leaves and roots under salt stress decreased back to initial levels.

### 2.3. FaHsfC1b-Transgenic Arabidopsis Exhibited Improvement in Heat Tolerance

**Figure 5:**
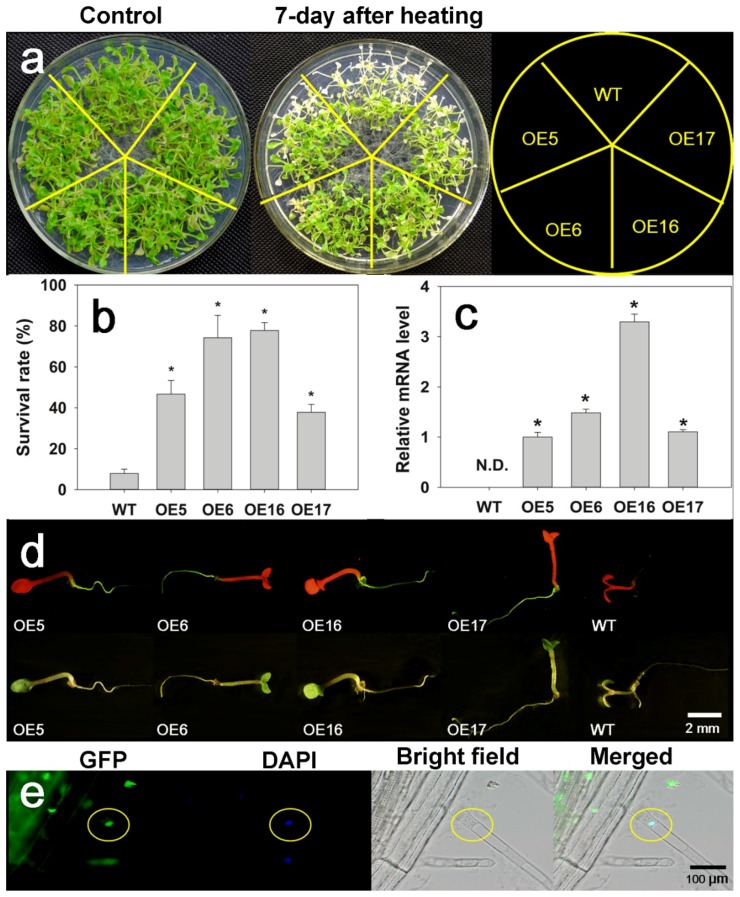
Physiological characterization of *FaHsfC1b*-transgenic Arabidopsis seedlings. (**a**) Showed three-week-old WT and transgenic Arabidopsis recovered from heat treatment (45 °C for 5 h) or grown in normal conditions. (**b**) Percentage of plants that survived after 7-day recovery from heat treatment. Values were means ± SD of five independent lines, fifteen seedlings in each dish for each line, and three biological replications. Asterisks on the top of bars indicated significant differences between WT and transgenic lines according to Fisher’s protected LSD test (*p* < 0.05). (**c**) Relative mRNA expression levels of *FaHsfC1b* in different lines. Values are means ± SD of five independent lines and three biological replicates (with three technical replications for each biological replication). N.D. indicated ‘not detectable’. (**d**) Expression of FaHsfC1b-GFP fusion protein in *FaHsfC1b* transgenic lines. Five-day-old seedlings in culture dish were observed under stereo fluorescence microscope. The upper row indicates seedlings under fluorescence light, while the lower row indicates seedlings under white light. (**e**) Localization of FaHsfC1b protein in root tips of transgenic plants. Five-day-old seedlings in culture dishes were observed under fluorescence microscope BX53 with higher magnification.

**Figure 6:**
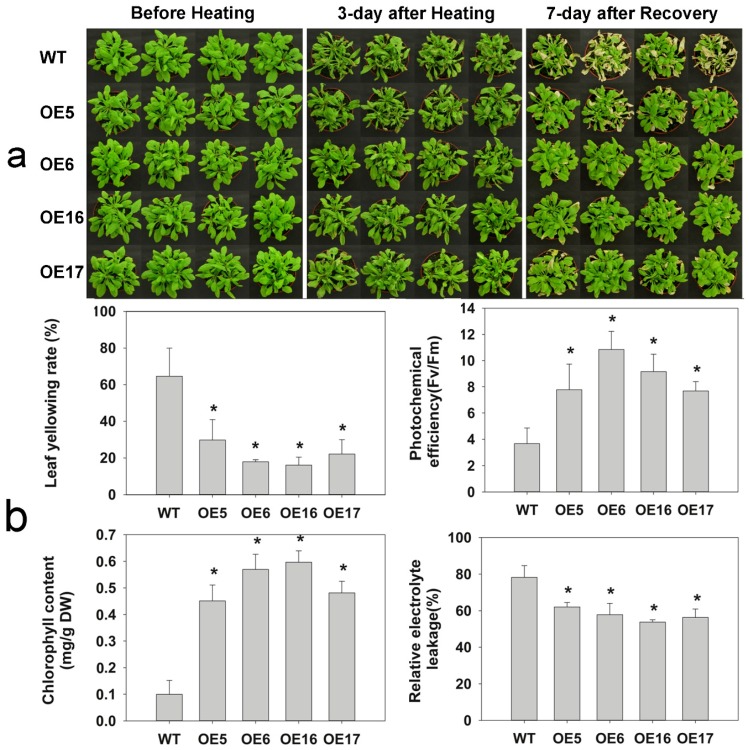
Heat tolerance of *FaHsfC1b*-overexpression Arabidopsis. (**a**) Performance of 6-week-old WT and transgenic plants in pots before heat treatment, after heat treatment (12-h 45 °C/12-h 40 °C), and after recovery (25 °C). (**b**) Leaf yellowing rate, photochemical efficiency (*F*_v_/*F*_m_), total chlorophyll content (Chl), and relative electrolyte leakage (EL) of plants in Figure 6a after 7-day recovery. Values were means ± SD of four different biological replications. Asterisks on the top of bars indicated significant differences between WT and transgenic lines according to Fisher’s protected LSD test (*p* < 0.05).

In order to investigate the function of FaHsfC1b, we transformed pEarleyGate103-*FaHsfC1b* into Arabidopsis by the Agrobacterium-mediated floral-dip method. Twenty T1 transgenic Arabidopsis lines were obtained by glufosinate ammonium selection and PCR validation (Figure S1). T2 seedlings and WT were exposed to 45 °C for 5 h. Four FaHsfC1b-overexpression transgenic lines which showed significantly higher survival rates under heat stress were selected for further analysis. The survival rates of the four transgenic lines, OE5 (with survival rate of 46%), OE6 (74%), OE16 (77%), OE17 (37%), were significantly greater than that of the WT (8%) (Figure 5a,b). The transgenic lines did not show any different phenotype from the WT under nonstress conditions (Figure 5a and Figure 6a). Expression of *FaHsfC1b* was detected in transgenic lines but not in WT, with OE16 having the highest expression (Figure 5c). GFP-fused FaHsfC1b protein was detected in roots of transgenic Arabidopsis but not in WT roots (Figure 5d). The protein was localized to the nucleus (Figure 5e). Tolerance to osmotic and salt stress was also examined on MS medium supplemented with mannitol and NaCl in petri dishes, and no significant differences in lateral root number and primary root length were detected between WT and transgenic lines (Figure S2).

The heat tolerance of four *FaHsfC1b*-overexpression lines and the WT were also evaluated for mature plants growing in substrate (Figure 6a). After 3 days of heat treatment at 45 °C/40 °C, transgenic lines had less severe leaf wilting compared with the WT. During a recovery period of 7 days at optimal growth temperature following heat stress, most of wilted leaves in the WT became yellow, whereas transgenic plants had lower leaf yellowing rates than the WT. Leaf photochemical efficiency (*F*_v_/*F*_m_) and chlorophyll content in OE lines were significantly higher than those in the WT. *F*_v_*/F*_m_ and chlorophyll content of OE6 and OE16 were about 6- and 3-folds higher, respectively, than those of the WT. Leaf relative electrolyte leakage (*EL*) in OE lines (about 60%) was significantly lower than that in the WT (80%) (Figure 6b).

**Figure 7:**
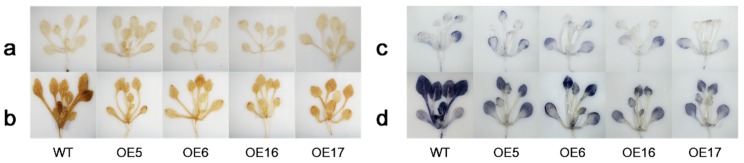
Detection of ROS of *FaHsfC1b*-transgenic Arabidopsis and WT. Plants stained by DAB (**a**) and NBT (**c**) under normal conditions. Plants stained by DAB (**b**) and NBT (**d**) after heat stress at 37 °C for 1 h.

The DAB and NBT stain assay was carried out to examine the production of H_2_O_2_ and O^2−^ of leaves in all lines grown under optimal temperature or heat stress at 37 °C for 1 h (Figure 7). Under the optimal growth temperature, there were no differences between the WT and transgenic lines (Figure 7a,c). Leaves showed a darker blue color in the WT compared to a light blue in OE lines (Figure 7d) and darker brown in the WT compared to light brown in OE lines (Figure 7b), demonstrating more O^2−^ and H_2_O_2_ produced in the WT than in the transgenic lines under heat stress.

### 2.4. Upregulation of Endogenous Erabidopsis Hsf1C Gene, Heat Protection Protein Genes, and ABA-Related Genes Associated with Overexpression of FaHsfC1b in Response to Heat Stress

**Figure 8:**
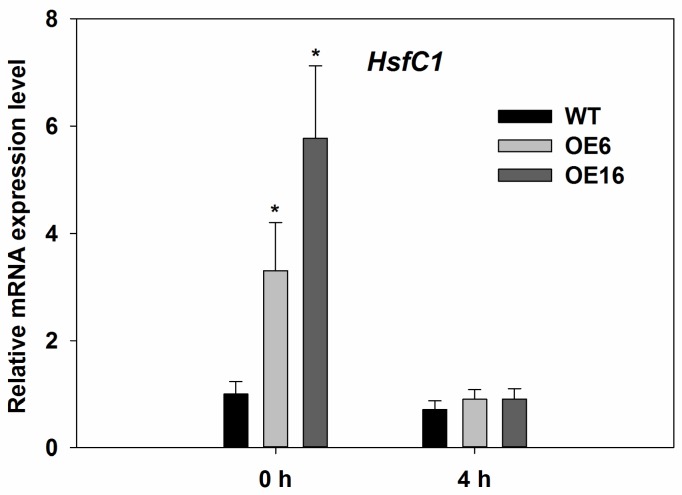
Relative mRNA expression level of endogenous *HsfC1* in WT and transgenic lines. Leaves of WT, OE6, and OE16 were collected under normal temperature and heat stress (37 °C) conditions for 4 h. Values were means ± SD of three different biological replications (with two technical replications for each biological replication). Asterisks on the top of bars indicated significant differences between WT and transgenic lines at each time period according to Fisher’s protected LSD test (*p* < 0.05).

In order to further confirm the roles of FaHsfC1b in plant adaptation to heat stress, the relationship between *FaHsfC1b* and endogenous Arabidopsis *HsfC1* was determined. *HsfC1* expression level in Arabidopsis was significantly upregulated in OE6 and OE16, as indicated in Figure 8, which suggests that endogenous *HsfC1* was activated by overexpression of *FaHsfC1b* in Arabidopsis. However, under heat stress for 4 h, *HsfC1* expression decreased to the initial level and no significant difference of *HsfC1* expression was detected between the WT and transgenic lines (Figure 8).

In order to further determine the underlying mechanisms for the positive effects of *FaHsfC1b* on heat stress, or specifically whether FaHsfC1b may induce or upregulate the expression of selected genes with known heat-protective functions, four HSP genes (*AtHsp18.1-CI*, *AtHsp22.0-ER*, *AtHsp26.5-P(r)*, *AtHsp70b*), *GalSyn1*, *AtRof1*, and *AtHSA32*, as well as four ABA-related genes, were compared between the WT and plants overexpressing *FaHsfC1b*. The gene expressions were analyzed in leaves but not in roots of *FaHsfC1b*-overexpressing transgenic plants, since *FaHsfC1b* expression was found to be induced earlier and more intensely in leaves than in roots in heat stress (Figure 4).

**Figure 9:**
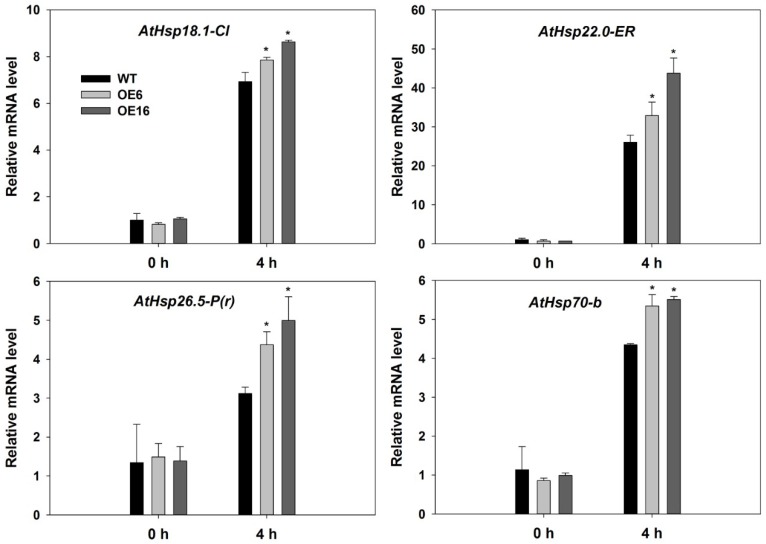
Relative mRNA expression levels of heat shock protein genes. OE6 and OE16 were selected to be tested in thermal environment (37 °C), and leaf samples were collected at 0 and 4 h after heat stress. Values were means ± SD of three different biological replications (with two technical replications for each biological replication). Asterisks indicated significant differences between means of WT and transgenic lines at each time period according to Fisher’s protected LSD test (*p* < 0.05).

Under optimal growth temperature conditions, no significant differences between WT and transgenic plants in the expression levels of all selected *HSP* genes were detected. In response to heat stress at 37 °C, the expression levels of *AtHsp18.1-CI*, *AtHsp22.0-ER*, *AtHsp26.5-P(r)*, and *AtHsp70b* were significantly elevated and higher in OE6 and OE16 than in WT (Figure 9).

**Figure 10:**
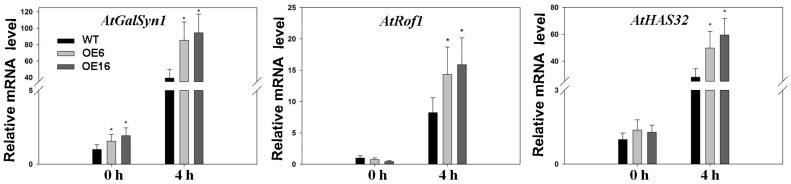
Relative mRNA expression levels of other heat protection genes. OE6 and OE16 were selected to be tested in thermal environment (37 °C) and leaf samples were collected at 0 and 4 h after heat stress. Values were means ± SD of three different biological replications (with two technical replications for each biological replication). Asterisks indicated significant differences between means of WT and transgenic lines at each time period according to Fisher’s protected LSD test (*p* < 0.05).

The expression level of *AtGalSyn1* was significantly higher in OE6 and OE16 than in the WT under normal temperature and heat stress conditions (Figure 10). The expression level of *AtGalSyn1* was further upregulated in both WT and transgenic plants in response to heat stress, and to a greater extent for OE6 and OE16 than the WT. *AtRof1* and *AtHSA32* did not exhibit differences in expression levels between the WT and transgenic plants under normal temperature, but were upregulated to a significantly higher level in OE6 and OE16 than in WT in response to heat stress (Figure 10).

**Figure 11:**
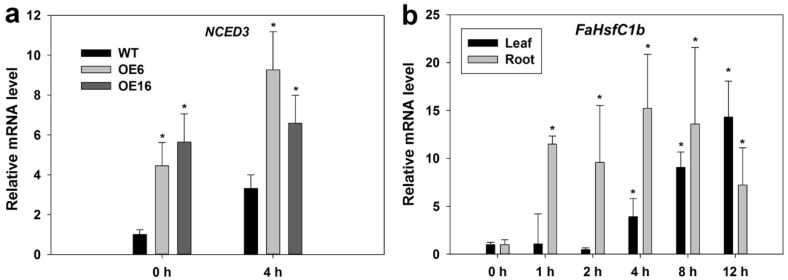
Relative mRNA expression levels of *NCED3* and *FaHsfC1b.* (**a**) Relative mRNA expression levels of *NCED3* in WT and transgenic Arabidopsis plants. OE6 and OE16 were selected to be tested in thermal environment (37 °C) and leaf samples were collected at 0 h and 4 h after heat stress. Values were means ± SD of three different biological replications (with two technical replications for each biological replication). Asterisks indicated significant differences between means of WT and transgenic lines at each time period according to Fisher’s protected LSD test (*p* < 0.05). (**b**) Relative mRNA expression levels of *FaHsfC1b* in leaves and roots under 100 μM ABA treatment in tall fescue. qRT-PCR values were means ± SD of three biological repetitions (three technical repetitions for every biological repetition). Asterisks indicated significant difference of mean values at each time period of stress treatment regarding the value at 0 h (nontreated plants) for each tissue analyzed (root and leaf).

For ABA-related genes, overexpressing *FaHsfC1b* lead to strong upregulation of the key gene involved in ABA biosynthesis, 9-*cis*-epoxycarotenoid dioxygenase 3 (*NCED3*), under normal temperature and heat stress (Figure 11a) but did not cause significant changes in the expression levels of *ABA deficient 1* (*ABA1*, codes for zeaxanthin epoxidase (*ZEP*)), *ABA2* (codes for short chain alcohol dehydrogenase/reductase (*SDR*)), and *ABA insensitive 1* (*ABI1*) that are involved in ABA biosynthesis (*ABA1* and *ABA2*) and signaling (*ABI1*) (Figure S3). In turn, *FaHsfC1b* expression was induced by ABA treatment in both leaves and roots in tall fescue (Figure 11b).

## 3. Discussion

As argued in the introduction, Class C Hsfs were less characterized compared to Class A and Class B Hsfs [22]. This study first identified and cloned a Class C Hsf in tall fescue (FaHsfC1b), which is located in the nucleus, suggesting that this gene is a putative transcription factor (Figure 1, Figure 2 and Figure 3). FaHsfC1b had a highly structured DNA binding domain located close to the N-terminus, a hydrophobic oligomerization domain, and a nucleus localization signal (Figure 1). The OD-dependent interactions were found to form hetero-oligomeric complexes between HsfA1 and HsfA2 and between HsfA4 and HsfA5 [11,42]. FaHsfC1b may interact with other Hsfs through OD-dependent manner, but this needs further investigation.

OsHsfC1b controlled plant development in addition to its function in abiotic stress, since retarded growth was observed in the *hsfc1b* mutant and knock-down lines under nonstress conditions [22]. However, transgenic Arabidopsis overexpressing *FaHsfC1b* did not exhibit phenotypic differences when compared with the WT. This may be caused by the plant’s heterologous system, since Arabidopsis is a dicot, while tall fescue is a monocot. Furthermore, only one *HsfC1* was presented in Arabidopsis, while tall fescue had several *HsfCs*, and each gene may function divergently.

Hsfs are known to play roles in stress adaptation for various plant species, but differential functions of different types of Hsfs may be involved with different stresses and stress severities. *HsfC1b* was previously showed to be upregulated by heat and many other environmental stresses [16,43,44,45]. In this study, *FaHsfC1b* was found to responsive to heat, cold, osmotic, and salt stress, which was similar to previous reports (Figure 4). The function of HsfC1b was only analyzed in rice by inserting a T-DNA sequence to disturb the expression of *OsHsfC1b*, and OsHsfC1b was found to be associated with plant tolerance to osmotic stress and salt stress, as demonstrated by shorter shoot and root length in mutants compared with the WT [22]. Unlike OsHsfC1b, overexpression of *FaHsfC1b* in Arabidopsis did not have significant effects on plant tolerance (no significant difference in primary root length and lateral root number between WT and transgenic lines) to osmotic stress and salt stress in our study (Figure S2). In wheat, *TaHsfC2a* was significantly upregulated by drought treatment, but transgenic plants showed no obvious increase in tolerance to drought stress [21]. Our findings showed the same phenomenon, however the underlying mechanisms need further clarification. To our knowledge, the physiological functions of HsfC1b in plant tolerance to heat stress have not been reported. In our study, to further investigate the functions of FaHsfC1b in heat tolerance, survival rates of seedlings and physiological traits of mature plants, including leaf yellowing rate, photochemical efficiency, chlorophyll content and membrane stability, as well as the production of relative oxygen species were analyzed in transgenic Arabidopsis lines overexpressing *FaHsfC1b*. Seedlings of transgenic lines exhibited significantly higher survival rate under heat stress and better recovery following heat stress compared with the WT (Figure 5). Furthermore, transgenic lines of mature plants exhibited significantly lower leaf yellowing rate and higher chlorophyll content and photochemical efficiency (Figure 6). Those results indicated that FaHsfC1b helped to alleviate heat damage to the photosynthetic systems. In addition, transgenic lines exhibited a lower degree of EL and H_2_O_2_ and O^2−^ production than the WT under heat stress, indicating the positive roles of FaHsfC1b in protecting plants from oxidative damage and maintaining cellular membrane stability (Figure 6 and Figure 7). Results from this study strongly suggested that FaHsfC1b conferred heat tolerance.

HSPs are known targets of Hsfs, as stated in the introduction. In this study, three sHsps (Hsp18.1-Cl, Hsp22.0-ER, Hsp26.5-P(r)) and one high molecular-mass proteins (Hsp70-b) were examined. All four *Hsp* genes were upregulated after heat treatment (Figure 9). Hsp18.1 was located in cytoplasm [46], and it was positively related to cellular thermotolerance and is used as a biomarker together with other Hsps (Hsp17.9A, Hsp17.7 and Hsp16.9A) for screening plants with superior heat stress tolerance [47]. The interaction of hetero-oligomeric complexes of Hsp18.1 and Hsp17.7 is essential for the formation of heat shock granules so that bound proteins are covered and protected under heat stress [48]. Promoters of Hsp18.1 and Hsp26.5 are able to bind to both HsfA1 and HsfA2, and co-expression of early (HsfA1a and HsfA1b) and late Hsfs (HsfA2) caused additive effects only when the induction of Hsp18.1 promoter occurred [29]. Hsp22 was located in endoplasmic reticulum and was found to have a high expression level in seeds and responded to heat stress in plants [49,50]. Hsp70 is essential for normal growth and contributes to plant tolerance to heat stress [39]. In this study, gene expression levels of *Hsp18.1*, *Hsp22*, *Hsp26.5*, and *Hsp70* were higher in transgenic lines of Arabidopsis (OE6 and OE16) overexpressing *FaHsfC1b* than that in the WT under heat stress (Figure 9), suggesting that the elevated expression of those *HSP* genes due to overexpression of *FaHsfC1b* could contribute to the improved heat tolerance of transgenic lines. Meanwhile, other heat protection genes like *AtGalSyn1*, *AtRof1* and *AtHSA32* also showed higher expression level in transgenic plants under heat stress (Figure 10). GalSyn participated in the synthesis of galactinol, which contributed to osmoprotection that has been proved to positively regulate plant thermotolerance [35]. Notably, *AtGalSyn1* was activated by overexpressing *FaHsfC1b* under normal temperature which is consistent with the endogenous upregulation of *HsfC1* in Arabidopsis (though HsfC1 was not induced at 4 h after heat stress and no difference between WT and transgenic plants was detected which may be caused by dynamic expression pattern of HsfC1 or regulation of unknown factors) (Figure 8). In wheat, TaHsfC2a could bind to HSE element in *TaGalSyn* promoter [21], but whether FaHsfC1b can directly activate *FaGalSyn* needs further study. As a novel HSP, HSA32 is heat stress inducible and crucial for thermotolerance during long recovery in Arabidopsis [32,33,34]. Rof1 interacted with HSP90.1 to modulate thermotolerance by affecting the accumulation of certain sHSPs [33]. All these results suggested that FaHsfC1b may function through regulating these heat protection genes to confer thermotolerance. However, the specific heat protection proteins serving as target genes for FaHsfC1b are still largely unknown, this study may be approached by RNA-Seq or microarray analysis in the future.

It is found that pre-treatment with ABA confers enhanced thermotolerance in monocot species like tall fescue, maize and creeping bentgrass (*Agrostis stolonifera*) [13,51,52]. Heat-stressed winter rape contained higher ABA content compared with the control plants [53]. ABA was found to be involved in heat stress in alga, as heat stress significantly increased endogenous ABA content in the alga [54]. Two ecotypes of reed (*Phragmites communis* Trin.), ‘dune reed’, and ‘swamp reed’ exhibited different tolerance levels to heat stress due to their differential ABA synthesis abilities under heat stress [55]. All these evidences suggested that ABA may play an important function in plant thermotolerance. The key enzyme NCED3 that is involved in ABA biosynthesis was significantly upregulated by overexpressing *FaHsfC1b* (Figure 11a). However, expression levels of another two genes involved in ABA biosynthesis (*ABA1* and *ABA2*) and one gene participating in ABA signaling (*ABI1*) showed no significant differences between WT and transgenic lines both under control and heat stress conditions (Figure S3). Meanwhile, exogenous ABA induced the expression of *FaHsfC1b* in tall fescue (Figure 11b). These results suggest that ABA may be involved in *FaHsfC1b* regulation of heat tolerance, but the relationship of ABA and *FaHsfC1b* deserves further investigation. One possible explanation is that FaHsfC1b and ABA interacted with each other to regulate plant tolerance to heat stress. Some HSP genes are regulated by ABA [56,57,58,59]. In wheat, TaHsfC2a acts downstream of ABA to directly or indirectly activate heat protection genes to enhance heat tolerance [21]. In tall fescue, *FaHsfA2c* and *HSPs* were upregulated by ABA which contributed to better heat tolerance [13]. OsHsfC1b was found to have a transactivation function to bind with the HSE element [60]. Since sequence alignment showed that FaHsfC1b had high similarity to OsHsfC1b, we postulated that these two genes may have similar binding characteristics. It is particularly interesting that FaHsfC1b may directly activate the expression of NCED3. Further research would be necessary to identify the binding elements between FaHsfC1b and the promoter of downstream target genes in order to better understand the molecular components of the regulatory network where FaHsfC1b is involved in contributing to tolerance to heat stress.

## 4. Materials and Methods

### 4.1. Plant Growth Conditions and Treatments

Tillers of tall fescue (cv. ‘Regenerate’) were collected from stock plants and planted in pots filled with a mixture of peat, vermiculite, and pearl stone (3:1:1 *v*/*v*/*v*). Plants were maintained in a greenhouse with natural sunlight and average day/night temperature of 25/20 °C. Plants were watered every three days and fertilized once a week during the seedling establishment.

For detection of relative expression levels of *FaHsfC1b* under different abiotic stress, tillers from stock plants were transplanted into to half-strength Hoagland’s nutrient solution [61] for two weeks in an environment-controlled growth chamber (MT8070iE, Xubang, Henan, China). The nutrient solution was changed once every week. Abiotic stress was carried out in hydroponic conditions. Plants were exposed to osmotic stress (20% polyethylene glycol 6000 solution (PEG-6000)), salt stress (150 mM NaCl), cold stress at 4 °C, or heat stress at 45 °C for 48 h. Each treatment had four biological replicates in four containers. All the treatments were conducted in environment-controlled growth chambers at a 12-h photoperiod with PAR of 500 µmol photons m^−2^·s^−1^. Roots and leaves were sampled at 0, 1, 2, 4, 8, 12, 24, and 48 h, and stored in a −80 °C freezer (Haier, Qingdao, China). 0.0264 g ABA powder was dissolved in 2 mL ethanol to make a 50 mM stock solution. For ABA treatment, ABA stock solution was added to half-strength Hoagland’s nutrient solution to reach a final concentration of 100 μM. Plant roots were subjected to ABA for 12 h, and leaves and roots were collected at 0, 1, 2, 4, 8 and 12 h.

For analysis of heat tolerance of transgenic Arabidopsis overexpressing FaHsfC1b, Arabidopsis ecotype Columbia (col-0) was used as the wild-type (WT). WT and T2 transgenic seeds were germinated on MS medium which contained 3% sucrose, 0.7% agar and supplemented without or with 20 μg·mL^−1^ glufosinate ammonium (Sigma, St. Louis, MO, USA). Both WT and transgenic seeds were placed in dark conditions at 4 °C for 3 days and maintained in the environment-controlled growth chamber controlled at 23/20 °C (day/night) with a 12-h photoperiod, and 70% relative humidity for germination.

For the heat stress treatment on seedlings of Arabidopsis in the MS medium, 5-day-old WT and transgenic plants were transferred to petri plates of MS medium in a growth chamber for 3 weeks and were then exposed to heat stress at 45 °C for 5 h followed by exposure to 23 °C for 7 days to examine post-stress recovery. For osmotic and salt stresses, one-week-old plants in MS medium were transferred to MS with 400 mM mannitol or 160 mM NaCl supplemented. Primary root length and lateral root number were measured for WT and transgenic plants (OE5, OE6, OE16 and OE17) after 14 days treatment.

For heat stress treatment on mature plants of Arabidopsis grown in substrate, four transgenic lines (OE5, OE6, OE16 and OE17) and the WT were transferred to pots filled with peat-vermiculite mix (3:1 *v*/*v*) for 6 weeks and then were subjected to heat stress at 45/40 °C (day/night) for 3 days followed by exposure to 23 °C for 10 days to examine post-stress recovery.

For qRT-PCR analysis of FaHsfC1b and downstream heat protective gene expression in response to heat stress, 3-week-old plants on MS medium were exposed to heat stress at 37 °C. Samples were collected after 4 h of heat stress treatment.

### 4.2. Isolation Fahsfc1b Gene from Tall Fescue

For gene isolation, 30-day-old tall fescue plants were subjected to 45 °C for 1 h in a growth chamber to induce *Hsf* gene expression. Total RNA was extracted from leaf tissues using the Plant RNA Kit (50) (Omega Bio-Tec, Norcross, GA, USA). RNA quality and integrity was checked by gel electrophoresis and Tecan Infinite 200 Pro (Austria GmbH, Grödig, Austria), and quantity was determined by Tecan Infinite 200 Pro. cDNA was synthesized using the PrimeScript 1st strand cDNA Synthesis Kit (Takara, Otsu, Japan). Primers were designed according to our transcriptome database of tall fescue using the software Primer Premier 5.0 (All primers used in this study were listed in Table S1). By using touch-down PCR [62], full length open reading frames (ORFs) were obtained. The PCR conditions were set as follows: the initial denaturation started at 98 °C for 30 s, followed with a succession of different cycles, 5 cycles of PCR (98 °C for 10 s, 70 °C for 30 s, 72 °C for 3 min), 5 cycles of PCR (98 °C for 10 s, 68 °C for 30 s, 72 °C for 3 min), 10 cycles of PCR (98 °C for 10 s, 65 °C for 30 s, 72 °C for 3 min), 15 cycles of PCR (98 °C for 10 s, 62 °C for 30 s, 72 °C for 3 min), and finally 72 °C for 10 min. The blunted-end fragment was then cloned into pJET 1.2/blunt vector. The inserted fragment was confirmed by colony PCR before samples were sent for sequencing (Springen Co., Ltd., Nanjing, China).

### 4.3. Subcellular Localization of Fahsfc1b Protein

Arabidopsis protoplasts were isolated using the method described by Wu et al., 2009. Large amounts of Arabidopsis protoplasts were obtained by peeling off the lower side of the epidermal cell layer and then digesting them in 15 mL enzyme solutions in light for 1 h. After washing with W5 solution, resuspended protoplasts were prepared for transient transformation. Linearized pENTR1A-FaHsfC1b had a LR recombination with p2GWF7.0 vector (~6.7 Kb) to construct p2GWF7.0-FaHsfC1b vector with eGFP fused at the C-terminal of the FaHsfC1b protein. Ten μg of the p2GWF7.0-FaHsfC1b plasmid were introduced into protoplasts by Polyethylene glycol 4000 (PEG-4000) (Fluka, Milwaukee, WI, USA) mediated method [63]. Followed by 16 h incubation at 25 °C under light in six-well plates, protoplasts were observed and photographed by a confocal laser scanning microscope (Carl Zeiss, Jena, Germany). For the transgenic Arabidopsis, root tips of 5-day-old seedlings were observed under a BX53 fluorescence microscope (Olympus, Tokyo, Japan). In the above two experiments, DAPI dyeing was performed to indicate the nucleus by adding DAPI solution to a 10 μg·mL^−1^ final concentration.

### 4.4. Plasmid Construction and Plant Transformation

pJET 1.2-FaHsfC1b was digested with EcoRI and EcoRV to get a FaHsfC1b ORF without a stop codon, which was then inserted into pENTR1A dual Selection Vector by T4 ligase. Linearized pENTR1A-FaHsfC1b was recombined with a pEarleyGate 103 plasmid using the LR Clonase II enzyme mix (Invitrogen, Carlsbad, CA, USA). The CaMV35S::FaHsfC1b construct was transformed into Arabidopsis by floral dip method [64]. T1 seeds were selected first on MS medium (3% sucrose, 0.7% agar, and 20 μg·mL^−1^ glufosinate ammonium), and germinated seedlings, supposedly the positive transgenic ones, were transferred into pots, followed by PCR detection using the primers for FaHsfC1b (reverse primer) and pEarleyGate 103 expression vector (forward primer). T2 seeds were collected for the following experiment.

### 4.5. qRT-PCR Analysis

For qRT-PCR, total RNA was extracted from samples using the Plant RNA Kit (50) (Omega Bio-Tec, Norcross, GA, USA) and was then reverse-transcribed to cDNA using the PrimeScript RT reagent Kit with the gDNA Eraser (Perfect Real Time) (Takara, Otsu, Japan). qRT-PCR was finished with the SYBR Green I Master reaction system (Roche Diagnostic, Rotkreuz, Switzerland). The operating procedure by Roche LightCycler480 II machine (Roche Diagnostic, Rotkreuz, Switzerland) for qRT-PCR was as follows: Pre-denaturation at 95 °C for 10 min, followed by 40 cycles (15 s at 95 °C, 15 s at 60 °C and 20 s at 72 °C), with data having been collected at 65 °C in each cycle. During qRT-PCR, in order to obtain more accurate data, there were three biological replicates for every treatment and each cDNA sample had three technical replicates. The data was standardized by the reference genes, *Fatublin* for tall fescue and *AtActin2* for Arabidopsis. Gene expression levels were calculated by the 2^−ΔΔ*C*t^ method [65].

### 4.6. Physiological Characterization of Transgenic and Wild Type Arabidopsis Under Heat Stress

For photochemical efficiency of leaves, *F*_v_*/F*_m_ was detected by a fluorescence induction monitor (OPTI-Sciences, Hudson, USA) after 30 min of dark adaption.

For cell membrane stability, relative electrolyte leakage (*EL*) was measured. Fully expanded leaves (0.2 g) were washed and immersed in 30 mL deionized water in a 50 mL centrifuge tube, in which leaves were wrapped in a Kim-wipe. Initial value of electrolyte leakage, *C*_i_, was collected after centrifuge tubes were shaken on a shaker for 24 h. Leaf samples were then autoclaved for 20 min at 121 °C, and *C*_max_ was collected after another 24 h of shaking at 180 r min^−1^. The data were measured by a conductance meter (Thermo Scientific, Baverly, MA, USA). The value of EL was calculated by formula: *EL* = (*C*_i_/*C*_max_) × 100.

For chlorophyll content, 0.1 g fully expanded leaves were clipped and immersed in 10 mL 95% ethanol in 15 mL centrifuge tubes. After incubating in the dark at 25 °C for 72 h, light absorption value was obtained by a spectrophotometer (GE Healthcare Life Sciences, Cambridge, UK) under 663 nm and 645 nm wavelength light. Leaves were dried at 80 °C to get their dry weight. The formula used to calculate chlorophyll content is as follows: *C* = ((13.95 × *A*_663_ − 6.88 × *A*_645_ + 13.95 × *A*_645_ − 7.23 × *A*_663_) × 0.01)/dry weight.

For phenotypic characterization, photographs were taken by single lens reflex camera (Nikon D5100, Bangkok, Thailand). Photographs of seedlings in MS medium were collected at 7 days after heat stress treatment while photographs of mature plants grown in soil were taken prior to heat treatment, 3 days after heat treatment, and 10 days after post-stress recovery. The rate of survival was determined for seedlings in MS medium following recovery from heat damage and the yellow leaves (with at least 1/3 of the leaf being yellow) of mature plants were counted following recovery from heat treatment.

### 4.7. Histochemical Detection of Oxidation Resistance

For detection of O^2−^, nitroblue tetrazolium (NBT) method [59] was used for reference. Leaves were sampled from 7-day-old WT and transgenic lines in MS medium before and after heat treatment at 37 °C for 1 h. Seedlings were carefully clipped from the medium and then immersed in 25 mM HEPES buffer (pH 7.6), which contained 0.1 mg mL^−1^ NBT (Sigma-Aldrich, St. Louis, MO, USA). After coloration for 2 h in dark conditions at 25 °C, NBT solution was changed with 85% ethanol in order to dislodge chlorophyll in plants, and after 24 h photographs were taken by stereomicroscope (Olympus, Tokyo, Japan).

Detection of H_2_O_2_ was based on the 3,3′-diaminobenzidine (DAB) method [66]. 0.1 mg·mL^−1^ DAB (Sigma-Aldrich, St. Louis, MO, USA) was dissolved in 50 mM CAT buffer (pH 5.0). Samples were selected as mentioned before. After coloration for 24 h in dark conditions at 25 °C, DAB solution was changed with 85% ethanol. Finally, photographs were taken.

### 4.8. Statistical Analyses

Data in all experiments were analyzed using the general linear model test, using the analysis of variance program of SAS (SAS 9.0, Cary, NC, USA). Mean values between different WT and overexpression lines or different treatments at different times were defined as significant when the value of Fisher’s protected least significant difference (LSD) was lower or equal to 0.05 probability.

### 4.9. Accession Numbers

Accession numbers of the proteins used in this study were as follows: FaHsfC1b (KY475613), BdHsfC1b (Bradi2g489), TaHsfC1b (AHZ44773), ZmHsf30 (GRMZM2G086), SiHsfC1b (Si002580m), OsHsfC1a (Os01g43590), OsHsfC1b (Os01g53220), OsHsfC2a (Os02g13800), OsHsfC2b (Os06g35960), HsfC1 (At3G24520).

## Supplementary Materials

Supplementary materials can be found at http://www.mdpi.com/1422-0067/19/9/2702/s1.
