# Supplementary material for: Characterization and Functional Analysis of FaHsfC1b from Festuca arundinacea Conferring Heat Tolerance in Arabidopsis
Source: Int J Mol Sci. 2018 Sep 11;19(9):2702. doi: 10.3390/ijms19092702 (PMC6163916; doi:10.3390/ijms19092702)
Supplement: Supplementary file 1 [file ijms-19-02702-s001.pdf]

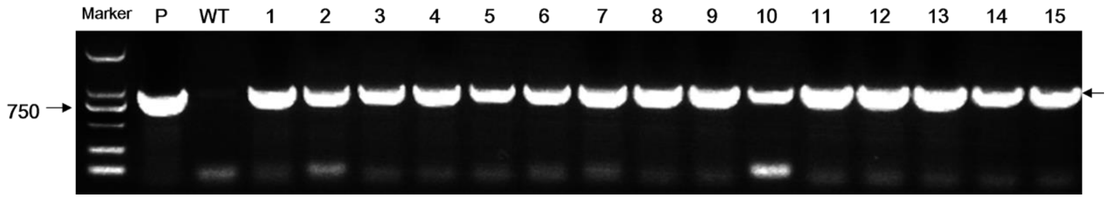

Figure S1. Detection of positive transgenic Arabidopsis lines. WT was set as negative control and pEarleyGate 103-*FaHsfC1b* was set as positive control. Only 15 positive lines were showed here.

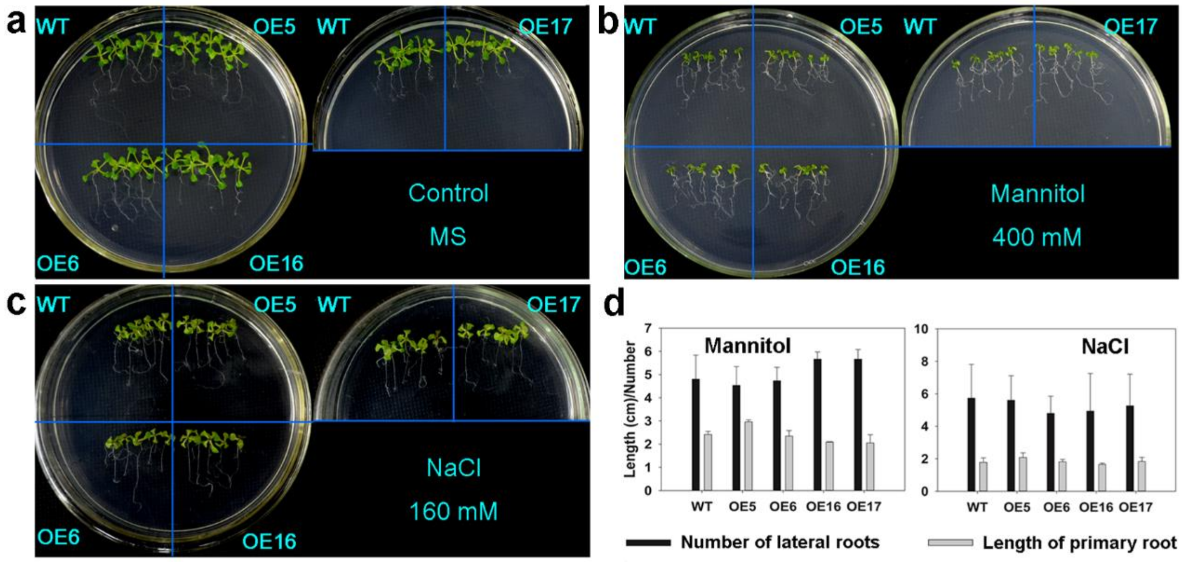

Figure S2. Physiological characterization of WT and FaHsfC1b-transgenic Arabidopsis seedlings under osmotic and salt stresses. (**a**) showed 3-week-old WT and transgenic Arabidopsis (OE5, OE6, OE16 and OE17) grown on MS medium in Petri dishes. (**b**) showed one-week-old WT and transgenic lines subjected to osmotic stress (400 mM mannitol) for two weeks. (**c**) showed one-week-old WT and transgenic lines subjected to salt stress (160 mM NaCl) for two weeks. (**d**) Number of lateral roots and length of primary roots in WT and four transgenic lines after two weeks subjecting osmotic and salt stress respectively. Values were means ± SD of five independent lines, five seedlings in each Petri dish for each line, and three biological replications. No statistically significant differences were found among samples.

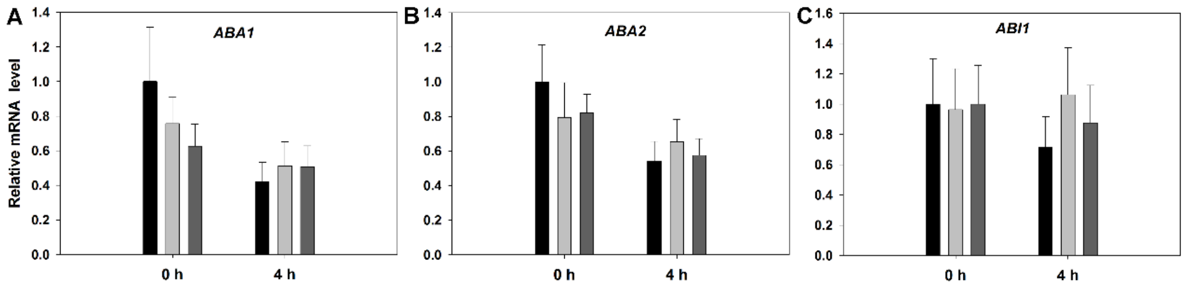

Figure S3. Relative mRNA expression levels of ABA signaling genes. OE6 and OE16 were selected to suffer in thermal environment (37 °C) and leaf samples were collected at 0 h and 4 h after heat stress. Values were means ± SD of three different biological replications (with two technical replications for each biological replication). No statistically significant differences were found among samples.

Table S1. Primers used in this study.

Primer name	Forward Primer Sequence (5′–3′)	Reverse Primer Sequence (5′–3′)
orfFaHsfC1b	GAATTCGCATGGGCAGCGAGTGCAAGG	GATATCTGTAGAACACTTGTCCCATAG
qFaHsfC1b	ACTTCAAGCACCGCAACTTT	TGAACCGGCCTTCTTCTTCT
qFaTublin	ATGCTTTCGTCTTATGCCC	CTCTTGGTTTTGATGGTTGC
qAtActin2	TGCCAATCTACGAGGGTTTC	TTCTCGATGGAAGAGCTGGT
qAtHsp18.1	GGGAAGTTTATGAGAAGGTTTAGGTT	CAAGCCAAGAAAAAAACACAAACT
qAtHsp22.0	GCTTGAGAATGGTGTGCTCACTAT	GGTAACTCTCTTCAGACTCAGAAAGTAATA
qAtHsp26.5	CAAAGAGTTATGGTTACTACAACACGA	ACGACACCGTATCTCTTCTACTCAA
qAtHsp70	GAAGAGGTGGATTAGAGCGTGTTTAGT	TTCATAACCTCTGGACCTTTGAGAC
qAtGalSyn1	TCTCGTGAATCTGAAGCCGT	GACCCGACACATAATCCCCA
qAtRof1	AAGGCCTTATACCGGAGAGC	TGCAACGTTTTCAAGACCTCA
qAtHSA32	CGTGGTCGTCATCTGGGTAA	TCTTACAGCATCAAAGAAGCACA
qHSFC1	TCTCTGTTTCGAGGTGGCTT	ACAAACGATGACTACTGACTCA
qAtNCED3	CGCCGTTAGCTTAGAGGTTG	GGGGACGTATATGCAGAGCA
qAtABA1	CCATCGATGCTTGACTGGGT	GTCTGCAACTAGGTGGCCTT
qAtABA2	ACTCGCTTTGGCTCATTTGC	ACAGAACAGCGTTCGCTACA
qAtABI1	CAGTGGAATGGAGCTCGTGT	TGCCATCTCACACGCTTCTT
